# A Case of Measles, with the Appearance on Dissection

**Published:** 1817-08

**Authors:** R. Morison

**Affiliations:** Assistant-Surgeon, R. N.


### *A Case of Measles, with the Appearance on Dissection.*

By Mr. R. Morison, Assistant-Surgeon, R. N.

February 11th, 1814. Jean Boucher, [a Frenchman], ætat. 28, a strong muscular man, of a melancholic habit, says, that he was seized yesterday morning with a fixed pain in the right side of the breast, which is worse to-day, and attended with a dry cough; hurried respiration; hot and dry skin; quick pulse; and a constipated state of bowels. Attributes his illness to cold. Measles prevail. Was bled immediately to thirty ounces, and a brisk cathartic afterwards given.

12th. Experienced temporary relief from the blood-letting; blood cupped, with a firm crassamentum. Cathartic operated well. Dyspnœa troublesome. Blood-letting to be repeated, and antimonial medicines to be given in the afternoon.

13th. Blood abstracted yesterday (20 ounces), exhibited inflammatory buff in a high degree; but the bleeding afforded little or no relief. This day a morbillous eruption appears on the face and neck; pain of the breast continues; pulse 98, quick, with a sharp feel; cough troublesome. Blood-letting repeated, and an antimonial cough mixture to be given. Six o'clock, p. m. Blood abstracted in the morning (20 ounces), and continues to shew much buff.

Bleeding repeated to 16 ounces, and a blister to be applied to the breast.

14*th, eight o'clock, a. m.* Passed a restless night. Blister rose well. Pyrexial symptoms increase in violence; tongue dry, and covered with a brown fur; no stool for the last twenty-four hours. To have a purgative; cough mixture continued.

*Six o'clock, p. m.* Purgative has procured two dark-coloured stools of an offensive fœtor; pulse continues quick, and with a sharp feel. Bleeding repeated to 24 ounces.

15*th, eight o'clock, a. m.* The bleeding last night was attended (as all the former bleedings) with no permanent good; the blood exhibited less buff. Morbillous eruption is disappearing; breathing more laborious, at times stertorous; pulse about 90, quick; skin very hot. Cough less urgent; perspires much during the day. Bleeding repeated to 16 ounces. Cough mixture continued.

*Six o'clock, p. m.* Now, for the first time, he expressed having experienced much relief from the blood-letting; vomited several times during the day. Medicine discontinued, and a blister to be applied between the scapulæ.

16*th, eight o'clock, a. m.* A bad night; blister rose well; countenance much depressed. Can only rest on the left side; continues to expectorate much thin aqueous sputum. Cough medicine continued.

*Six o'clock, p. m.* The vomiting returned this day; has had several loose stools; is sinking.

17*th.* No recurrence of the vomiting; bowels continue open; is at times comatose, and is fast sinking.

18*th.* This morning, at six o'clock, he expired.

DISSECTION. *Seventeen hours post mortem.*

Upon laying open the cavity of the thorax, no appearance of disease could be discovered; on the contrary, he had fine large healthy lungs. On cutting into their substance, the same universal healthy structure presented itself; the heart and large blood-vessels seemed sound; no liquor pericardii.

Laying open the cavity of the abdomen, the superior surface of the left lateral lobe of the liver adhered firmly to the contiguous part of the diaphragm; so much so, that it could not be separated but by dissection; the volume and structure of the liver itself appeared natural; the spleen felt rather firm. On examining the intestines, five progressive intus-susceptions were discovered in the course of the jejunum. The smallest possible force liberated the strangulated portions; the gut was of a dark hue in these places, and in the intervening spaces some light coloured bile appeared.

The stomach and other intestines were filled with air. No other morbid phænomenon could be discovered.

*Remarks.* There was something, I think, very mysterious in this case. Having had much experience in pulmonic complaints, I thought I could not be mistaken in the organ affected; for I never, in any instance, saw stronger or more unequivocal marks of determination to the respiratory organs, than this man exhibited from the first day of his illness till the day before he expired. The symptoms, the age, and constitution of my patient; the inflammatory nature of the disease; the good effects which I saw result from venæsection in other cases of measles now prevalent in the ship; all induced me to persevere in the depletory plan, and to think that I had not yet reached the foundation of the disease. If it be thought that I carried venæsection too far in this case, I answer, that having forty measle patients, principally adults, at this time, among whom I carried depletion much farther, both before and after this accident, without losing a man, I conceive I am authorised in attributing the death of my patient to the intus-susceptions, and the healthy *structure* of the lungs *post mortem*, to the active system of depletion during his illness. That diseased *action* was going on in the lungs from great determination of blood to those organs, I can have no manner of doubt; and had the treatment been less decisive, I have little hesitation in affirming, that morbid structure in the chest would have been developed on dissection. I trust, therefore, that the case offers, altogether, an instructive lesson; and instead of militating against rapid and decisive depletions in morbillous affections of the lungs, offers a most exemplary proof of their power in averting *structural* derangements, however pressing and alarming may be the *functional* disturbance.

I have had considerable experience in typhus fever, and have uniformly observed the special good effects of early and judicious bleeding in this disease. In four fatal cases of typhus, which had resisted this treatment, but which were protracted to a considerable period, [from 20 to 26 days] I inspected the bodies very carefully, but not a vestige of diseased *structure* could I find in any part. I believe this would rarely be the case in typhus dissections where the lancet and other means of unloading the vessels had been neglected, or only sparingly employed. In these instances, it seemed as if the *functional* derangements of the various organs wore out the thread of life, although the remedial aids of depletion, &c. had preserved them from any visible lesions of structure.

ROBERT MORISON.

March, 1817.

---

